# Experimental Demonstration and Circuitry for a Very Compact Coil-Only Pulse Echo EMAT

**DOI:** 10.3390/s17040926

**Published:** 2017-04-22

**Authors:** Dirk Rueter

**Affiliations:** Institute of Measuring and Sensor Technology, University of Applied Sciences Ruhr-West, 45479 Muelheim, Germany; dirk.rueter@hs-ruhrwest.de; Tel.: +49-208-88-254-388

**Keywords:** EMAT, pulse power, contactless ultrasound transduction, Lorentz forces, ultrasound echo recording

## Abstract

This experimental study demonstrates for the first time a solid-state circuitry and design for a simple compact copper coil (without an additional bulky permanent magnet or bulky electromagnet) as a contactless electromagnetic acoustic transducer (EMAT) for pulse echo operation at MHz frequencies. A pulsed ultrasound emission into a metallic test object is electromagnetically excited by an intense MHz burst at up to 500 A through the 0.15 mm filaments of the transducer. Immediately thereafter, a smoother and quasi “DC-like” current of 100 A is applied for about 1 ms and allows an echo detection. The ultrasonic pulse echo operation for a simple, compact, non-contacting copper coil is new. Application scenarios for compact transducer techniques include very narrow and hostile environments, in which, e.g., quickly moving metal parts must be tested with only one, non-contacting ultrasound shot. The small transducer coil can be operated remotely with a cable connection, separate from the much bulkier supply circuitry. Several options for more technical and fundamental progress are discussed.

## 1. Introduction

Electromagnetic acoustic transducers (EMATs) are contactless ultrasound transducers for metallic test objects [1]. Electroacoustic coupling is provided over a distance via magnetic fields and, practically interesting, is insensitive to non-metallic layers or impurities on the target metal’s surface (oil, dirt, water, oxides, or paint). In addition, a non-contacting EMAT is compatible with rapidly moving or very hot test objects. The typical disadvantages of an EMAT include poor electroacoustic conversion efficiency for emitting and receiving ultrasound signals and a limited range for magnetic coupling between metal and an EMAT, typically about 1 mm or even less.

**Figure 1:**
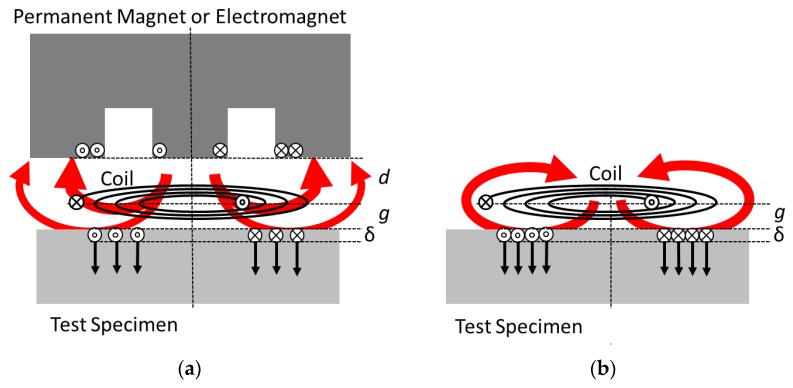
(**a**) A conventional EMAT includes a flat induction coil and a permanent magnet. The RF induction coil induces eddy currents (small circles) within the surface sheet (δ) of the nearby (distance g) metallic test object. The static field (red arrows) from the permanent magnet (in distance g + d) and the eddy currents affect the RF Lorentz forces (black arrows) within the skin sheet and thus excite ultrasonic vibrations in the test metal. Notably, the induction coil also induces eddy currents in the metallic parts of the magnet, which in return reduce the intended eddy currents in the target. (**b**) Without a permanent magnet, this decrease in the eddy currents in the target is absent. Here, the RF magnetic field of the induction coil itself—together with the eddy currents in the surface sheet—excite the ultrasonic vibrations in the test metal. Furthermore, the projected field from the induction coil and the spatial distribution of the eddy currents in the target are geometrically matched.

A conventional EMAT usually consists of a radio frequency (RF) induction coil and a bulkier permanent magnet or electromagnet [2] (Figure 1a). For ultrasound excitation in a nearby metallic target, the induction coil excites the RF eddy currents in the surface sheet of the metal and within the characteristic electromagnetic skin depth δ. Together with a DC magnetic bias field from the additional magnet, the metal surface experiences RF Lorentz forces. These RF forces or pressures are proportional to the eddy currents and to the present bias field. Similar to [3], this paper concentrates on Lorentz forces although other transduction mechanisms, such as magnetostriction, may apply for ferromagnetic materials [4,5,6]. In this demonstration study, however, only non-magnetic aluminum metal was used as the test specimen. The ultrasound intensity in the aluminum metal is proportional to the square of the RF Lorentz forces; thus, it is proportional to the square of the present bias field and the induced RF eddy current in the target plane. For reasonably strong ultrasound signals, therefore, it is desirable to drive strong RF currents through the induction coil and to provide a strong magnetic bias field for the target area. However, the available flux density from conventional permanent magnets is typically not much higher than 1 T, and the field strength strongly decreases over distance, which then directly affects the EMAT’s electroacoustic conversion efficiency or signal quality. As an additional and inherent disadvantage of conventional EMATs, the required RF induction coil and the nearby permanent magnet tend to obstruct each other (Figure 1a). Typically, a flat induction coil is arranged parallel to the metallic target surface and separated by a specific air gap g, i.e., the desired EMAT application as a contactless sensor over a distance g. A bulkier permanent magnet must be positioned beyond the induction coil and at an additional distance d. The total distance (d + g) reduces the flux density of the DC bias field in the target plane. The undesired decrease can be gradually overcome with a larger permanent magnet [3]; however, this solution is not suitable in every application. The permanent magnet’s materials (or the metallic windings of an electromagnet) tend to weaken the effect of the RF induction coil: induced RF eddy currents in a metallic permanent magnet (or in the windings of an electromagnet) are typically in counterphase with respect to the induction coil. Such additional eddy currents at the magnet’s site reduce the actual intended eddy currents in the metallic target. Furthermore, undesired ultrasound vibrations are excited in the nearby magnet, and they could interfere with the target’s echo signal. Thus, a tradeoff exists between positioning a permanent magnet as close as possible to the target (Figure 1a : d → 0) for the strongest possible DC bias field in the target plane and placing a metallic permanent magnet more distant from the induction coil (d > g) to preserve the available coupling efficiency and signal quality of the RF induction coil.

Contactless ultrasound excitation in metallic test objects is also possible with a simple compact induction coil alone (Figure 1b), without a permanent magnet [5,6,7,8,9,10,11]. As the transduction effect is proportional to the RF eddy current and the exciting RF induction field itself (“self-biasing”), the RF Lorentz forces follow a quadratic characteristic. Then the affected acoustic pressure becomes proportional to the square of the induction current, or in more detail, equals the energy density (=magnetic pressure) of the RF induction field at the metal target’s surface [10]. Consequently, because the ultrasound intensity is proportional to the square of the acoustic pressure, the acoustic intensity increases with the fourth power of the RF induction current. Therefore, a preferably very strong and pulsed excitation current is applied to the simple coil transmitter without an additional magnet. Beyond certain power levels, the RF flux densities can become higher than the practically available flux density from a permanent magnet, and then the emitted ultrasound intensity can readily outperform conventional EMATs. Intense electrical pulses and magnetic flux densities with amplitudes of more than 10 kV, 10 kA, and about 10 T have been reported, resulting in strong ultrasound intensities in the target up to kilowatt levels [10]. High-voltage capacitors and a spark gap switch provide the microsecond high-power pulses for this simple compact coil transmitter. This kind of transduction preferably excites normally oriented body forces, such as hydrostatic pressure, into the target, more suitable for generating longitudinal ultrasound waves in the volume and the near surface Rayleigh waves [8] in the lateral direction. A more detailed discussion about these pressure fields is given in [11] and is not extensively recapitulated in this practical demonstration. However, the scheme is less effective for exciting lateral body forces for shear wave emission into the bulk material. Suggestions for generating shear waves with the proposed technology are provided in the discussion.

As another and sometimes practically relevant advantage, without a permanent magnet, a simple coil transducer will not attract ferromagnetic particles from the environment. These adhering particles could affect the electroacoustic function or might even cause mechanical damage in an industrial EMAT application with nearby moving test objects.

However, a high-voltage, spark gap-controlled EMAT system is generally not very suitable for commercialization and widespread applications, because—in addition to expenditures for high-voltage equipment—spark gaps do not switch very reliably and, in particular, are not maintenance-free over a reasonable service period. A potentially maintenance-free reliable all-solid-state technology for a coil-only EMAT transducer is, wherever possible, mandatory. A solid-state design without high voltage and a spark gap is one approach of this contribution.

Another novel approach of this work is the demonstration of ultrasound echo detection with only a single simple coil. This approach has not been explicitly shown before, although it was predicted in a previous work [11]. The echo operation (contact-less ultrasound emission into a test object and echo detection) of a conventional EMAT with a permanent magnet is well-known and has been commercialized for many years. In contrast, a single inductor without a magnetic bias field cannot detect the ultrasonic vibrations of a nearby metal surface. Nevertheless, the inductor can be biased with a strong and, e.g., DC-like current pulse. Thus, the induction coil itself provides the required bias field. The ultrasonic vibration of a metal, when exposed to such a magnetic bias field, excites the RF currents in the metal’s surface with the characteristic ultrasound frequency. These RF currents induce an RF signal in the nearby coil. The typically small RF signal in the coil can be separated from the DC current, and ultrasonic vibrations can be detected in the nearby metal. This was briefly described in Ref. [11] but without a demonstration of the echo operation for a single coil. With a sufficiently intense magnetization current, an even stronger bias field can be provided than possible with a permanent magnet in a conventional EMAT. Helpful for the flux density and schematically clear from Figure 1a,b, the induction coil is positioned closer to the target (at a distance g and not d + g). Furthermore, the bias field geometry, naturally, is geometrically matched to the inductor’s spatial sensitivity. This is not necessarily the case for an inductor and a separate permanent magnet (Figure 1a), with deviating field topologies. The potentially high flux density and the matching field geometries promote the principal sensitivity of the receiver.

The aim and novelty of this practical contribution is to: (a) demonstrate contact-less ultrasound emission and subsequent ultrasound echo detection within a single, simple, compact copper coil without a permanent magnet or a separate electromagnet; and (b) modify the required setup and circuitry in a potentially maintenance-free solid-state design, without the disadvantages of high voltage and spark gaps. In addition, this transducer coil should be operational through a cable and remote from much bulkier (and more sensitive, not suitable for a narrow and hostile environment) supply electronics.

Thus, and for the first time, the explicit functionality of a single small, simple copper coil as a more compact, rigid, non-contacting, non-magnetic (no permanent magnet), and potentially maintenance-free ultrasound echo system is demonstrated. The circuitry with only one switching transistor is obviously scalable; therefore, much higher power is still possible. This is relevant when considering the quadratic non-linearity of the ultrasound emission.

Within the scope of this initial and intentionally lucent demonstration, however, it is not possible to optimize or tune the many and mutually interacting parameters, parts, and circuitry of the whole system for a fully mature or quasi-commercial performance. Therefore, further engineering, simulation, and circuit design should result in more progress. Suggestions for further progress and modifications are provided at the end of this work. The features in whole or in part might also be useful for further improvements in conventional EMAT setups.

## 2. Materials, Methods, and Circuitry

**Figure 2:**
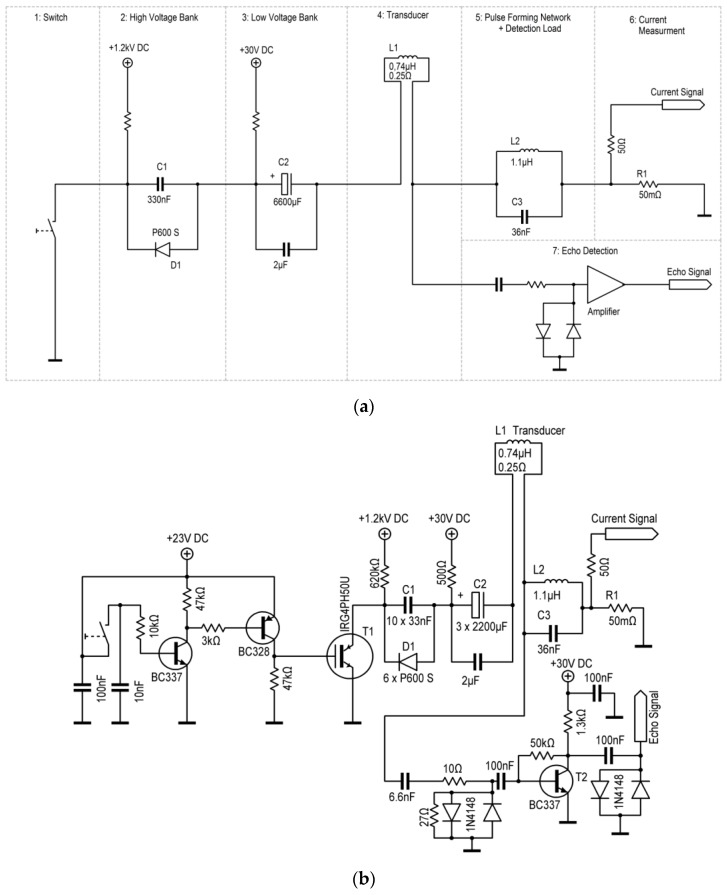
(**a**) The circuitry is based on seven blocks, each with a distinct function and influence. Pulsed and high currents/voltages occur in a serial connection from blocks 1 to 6. Block 4 is a small ultrasound transducer coil with (undesired but unavoidable) resistance of 0.25 Ω. The transducer can be connected to the circuitry with a cable. (**b**) The detailed circuitry contains only two relevant transistors: the insulated gate bipolar transistor (IGBT) T1 works as a fast high-power switch (block 1) and T2 as a simple amplifier (block 7) for the ultrasound echoes.

**Figure 3:**
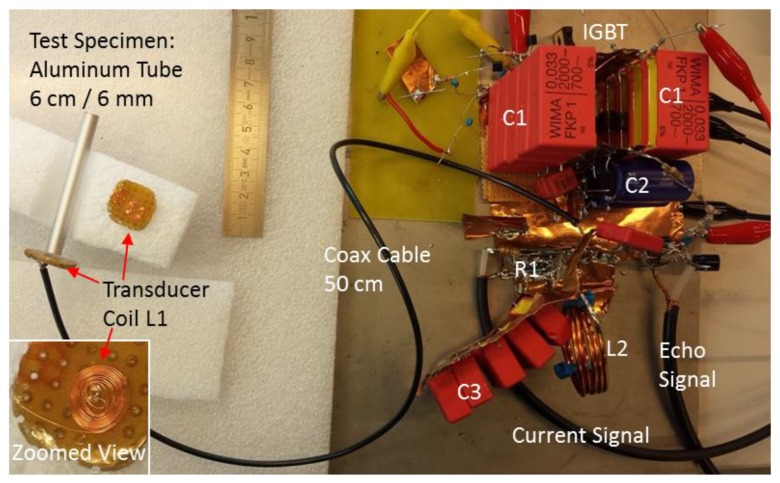
Practical appearance of the setup. Parts are labeled according to Figure 2. The small spiral coil was made from a 0.15 mm copper wire (magnified inset in the left bottom corner) and was repetitively pulsed up to 500 A from the circuitry. The coil magnetically excited and received megahertz ultrasound pulses from the test specimen, separated by a small air gap (typ. <1 mm).

The functionality of a simple coil transducer was established in the circuitry. Major attention has been focused on the electrical features and functionalities. Although each component or local arrangement in the circuitry is conventional, the functionality of the whole is new for this application. The system’s circuit consists of seven blocks with different tasks, which are described separately. In this design, blocks 1–6 are series connected and, therefore, strongly influence each other. Figure 2a shows the seven discrete blocks in their schematic function and Figure 2b the detailed circuitry. The photographs in Figure 3 reveal the actual dimensions of the setup: the transducer coil is much smaller than the supply circuitry. A cable connection is mandatory to utilize the small size of the transducer.

The transducer coil (function block 4 in Figure 2a or inset in Figure 3, literally in the center of the action) is a handmade flat spiral coil (“pancake coil”) with an 8 mm outer diameter and ten dense windings of an ordinary 0.15 mm coil wire (copper). The spiral windings are embedded in epoxy resin (consumer-grade glue, sets rapidly within 5 min, heat sensitive) and covered with a 0.125 mm Mylar film, which provides electrical insulation and mechanical protection for the spiral’s surface. Relevant electrical properties of the coil are (undesired but unavoidable) resistance of 0.25 Ω and free space inductance of 0.74 µH. The inductance can be determined by measuring the LC resonance with a parallel capacitor. The epoxy glue is (intentionally) heat sensitive. It notably changes color from clear white to brownish when exposed to more than 150 °C over more than a minute. A changed color indicates critical temperature stress of the small coil. The total copper mass of the coil is about only 35 mg, and together with the heat capacity of copper (385 J kg^−1^ K^−1^), the coil temperature rises about 75 K at 1 J dissipated energy from a short (10 µs) electrical pulse. For a prolonged current pulse (here, 1 ms), heat conduction toward the immediate surroundings of the thin wires becomes effective, and then the copper’s temperature rise is significantly less than 75 K at 1 J dissipated energy.

The transducer coil can be operated through an ordinary two-wire cable (about 1.5 mm^2^ copper cross-section for each wire) or through a standard coaxial cable (50 Ω). With such an almost arbitrary cable connection and its inner serial inductance, resistance, and parallel capacitance, the overall system’s performance decreases somewhat, but the essential functionality remains conserved.

The energy and power for ultrasound emission are provided by a high-voltage capacitor bank (function block 2), ten parallel 33 nF capacitors, type FKP1 WIMA (pulse enhanced, 2000 V DC). This bank is charged up to +1200 V DC over a sufficiently dimensioned 620 kΩ resistor. At 1200 V, about 0.25 J of energy is stored in the bank. The 0.25 J can be quickly released into the small transducer coil, and, thus, the power densities still become high. The “only” 1200 V DC is easy to handle and has much less delicate insulation problems or undesired breakdowns. However, only experienced engineers should work with charged high-voltage capacitors, as they can cause serious electrical shocks. A single 0.25 J pulse from the high-voltage bank cannot critically heat up the copper coil; see the heat capacity considerations. In theory, when ideally switching an ideal 330 nF capacitor at 1200 V to an ideal 0.7 µH inductor, the initial current will rise within 0.7 µs to 750 A. Similar rise times and amplitudes have been observed in practice and simulated for the real transducer system, and—the purpose here—transient and strong magnetic fields and Lorentz forces occur close to the windings of the small coil. Although the field topology, the achieved flux density, and the forces between the coil and the target can be computed with a quasi-analytical method [11], a much more rough and simple calculation [10] suffices for this practical work: about 50% of the available electrical energy in the capacitor (here: 0.125 J) can momentarily convert into magnetic energy within the gap between the coil and the target metal. The volume of a 0.5 mm gap with an 8 mm diameter (the diameter of the coil) is close to 25 mm^3^, and then the momentary magnetic energy density *E_m_* in the gap becomes about 5 MJ/m^3^. At the same time, this magnetic energy density is mechanically effective on the target as transient pressure [5,6] with 5 MJ/m^3^ = 5 MPa (equivalent to 50 atm). The momentary magnetic flux density B in the gap is then, since $E_{m}=\frac{B^{2}}{2\mu_{0}}$, close to 4 T. This field is considerably stronger than that reported for standard EMATs with permanent magnets or electromagnets [2], and it becomes comparable to the recently described core-magnet arrangement for EMATs, which is based on a very sophisticated and strong (>3 T reported) but voluminous arrangement of permanent magnets [3]. This estimation of 4 T, although very simple, has been proven to be sufficiently reasonable [10], in which magnetic energy densities even up to 400 atm (equivalent to about 10 T) were calculated and experimentally confirmed.

A second capacitor bank with 3 × 2200 µF and 50 V electrolytic capacitors (low ESR, high-endurance type) is charged to +30 V (block 3). The 30 V supply must be carefully decoupled (using a sufficient resistor and an inductor) from this bank, as very transient strong signals from the high-voltage capacitor are present after switching, endangering the 30 V power supply and the insulated gate bipolar transistor (IGBT) switch itself (described below). When connecting only this low-voltage bank to the transducer coil, a prolonged current pulse with initially about 100 A (≈30 V/0.3 Ω) and an exponential decay (τ ≈ R ˑC ≈ 1.5 ms) is obtained. The smooth 100 A on the millisecond scale is almost DC-like with respect to the microsecond pulse from the high-voltage bank. This 100 A generates the required magnetic bias field for ultrasound detection. The energy content of this bank is about 3 J, thus much higher than in the high-voltage bank. About 2.5 J of this energy will convert into heat of the thin transducer coil windings and within about 1 ms. Therefore, the winding’s temperature will rise about 100 K (this estimation includes heat conduction to the immediate surroundings). For a single pulse, the temperature load of the coil is believed to be still below critical levels of about 200 °C. Discoloration of the embedding epoxy resin or any degradation of the coil was not observable after hundreds of shots with low repetition frequency. Nevertheless, a higher repetition frequency (>1 Hz) of the demonstration system is prohibited by the coil’s temperature stress. Suggestions for achieving significantly higher repetition frequencies are given in the discussion.

In this study, the low-voltage (block 3) and high-voltage (block 2) banks are merely series connected. A number of parallel, high-voltage, cost-effective, and high-current silicon diodes (P 600 S, 1200 V, 400 A peak) bypass the low-voltage current from the low-voltage capacitor around the high-voltage capacitor. These bypass diodes also result in a unipolar characteristic of the high-voltage pulse, as will be shown. Conversely, some parallel high Q capacitors with 2 µF in sum bypass the much more transient and stronger high-voltage current around the electrolytic capacitor.

An additional component (block 6) in the circuitry is the shunt resistance of 50 mΩ with 50 Ω terminal impedance, which serves here as a time-resolved current measurement. The 50 mΩ are obtained from 20 parallel 1 Ω resistors (standard types, 1/4 W), which then obtain negligible inductance and can carry strong current pulses. It is ensured that a 1 A current actually and instantaneously (nanosecond scale) results in a 50 mV measurement signal for a standard oscilloscope. Consequently, a 5 V signal in the scope indicates a current of 100 A.

A single cost-effective IGBT type IRG4PH50U (“Ultrafast and hard switching”, Collector-to-Emitter Voltage maximum 1200 V, Pulsed Collector Current maximum 180 A, Gate-to-Emitter Voltage maximum 20 V) serves as a switch, function block 1. In this study, only the switch functionality “Fast Turn-On” is required. This IGBT is rated with a rise time of 15 ns. The fall time (“Turn-Off”) is more prolonged, rated at 280 ns. The author believes that the internal losses and stresses are lower in the exclusive “Switch-On” operation. Then considerably higher currents can be tolerated from the IGBT over a short period of time. Here, almost 700 A at the 1200 V capacitor voltage is repeatedly (more than 100 times) observed without noticeable failure or degradation. The IGBT has the potential to serve as a maintenance-free solid-state switch for the intended application. What is important here is that a strong gate signal for such switching is necessary: a fast rise from 0 V to about 22 V (10% more than officially rated for the gate) with a low impedance signal is required, as shown with the PNP transistor in the gate control circuit in Figure 2b. A number of such IGBTs could be combined either in parallel or in series, and a carefully designed IGBT battery promises even higher power and current. A prolonged current pulse significantly above 1 kA from a parallel IGBT battery was described in a previous work [11]. Other IGBT types or solid-state switches might be even more suitable for the application, but they are currently not known to the author. In this study, only a single IGBT (IRG4PH50U, International Rectifier, El Segundo, CA, USA ) is utilized as a fast, cost-effective, high-voltage, high-current switch, and it restricts this demonstration system to more moderate power and current. At the same time, such restriction demonstrates the lean cost-effective feasibility of the proposed technology.

The influence of function block 5 with L2 and C3 is more complex. Without this LC combination and when the switch is closed, both ends of the transducer coil would be tightly connected to the ground regarding an induced megahertz signal from an ultrasound echo. Then, the echo signal would be short-circuited; it could not be tapped somehow. The inserted LC combination represents a load for such a detected echo signal. As an LC parallel circuit, it could be assumed to preferably select signals close to its resonance frequency. The 1.1 µH coil is handmade from thick (2 mm) copper wire, as no additional serial resistance (except the unavoidable 0.25 Ω of the small transducer coil and the 0.05 Ω for the current measurement) is desirable in the serial power chain from blocks 1–6. Together with capacitor C3, a resonance at about 0.85 MHz is calculated. With a closed switch, however, the inductances L1 and L2 are in parallel connection for the signals (for both inductors: one contact at C3 and the other one grounded). The joint inductance is then about 0.45 µH, which results—together with C3—in a frequency of about 1.25 MHz. This frequency and not 0.85 MHz dominates in the resulting signals, either emitted or received. Building block 5 does not virtually hinder or influence the slow 100 A discharge from C2. However, the block strongly impinges on the much faster discharge from C1. A considerable—and apparently helpful—megahertz modulation at 1.25 MHz is imprinted on the transient microsecond discharge, at levels of several hundred amperes and directly translating into transduction of an ultrasonic megahertz burst from the transducer coil. Thus, the functionality of block 5 strongly influences the high-power signals for ultrasound emission and the small signals of ultrasound echo detection.

Finally, the tapped echo signal is amplified by block 7, here intentionally realized as a simple and inexpensive transistor amplifier, which emphasizes the overall feasibility and transparency of the technique. The voltage amplification of this single-stage amplifier is about 100 at 1 MHz. This single-stage transistor amp with a collector operation point at about 5 V and 20 mA is suitable for detecting small signals at low impedance levels. Surprisingly, the transistor is sufficiently protected against the very strong emission pulses via only a capacitor, then 10 Ω and then two small and antiparallel clamping diodes together with 27 Ω for faster recovery and dampening. The amplifier stage should quickly recover after the strong emission pulse to immediately pick up the echo signals with small amplitudes, typically below 1 mV. Although the amplifier in this intentionally lucid demonstration system is only passively decoupled from the strong emission pulse, the recovery time for echo detection can be tuned down to 25 µs. Such recovery time already approaches the principal limit in this setup, since the strong emission pulse itself lasts for about 10 µs. The equivalent noise voltage of such a standard bipolar transistor at a low impedance operation point is usually much smaller than 10 nV/(sqrt Hz), and the intrinsic noise level of the amplified output would not be higher than 2 mV at 1 MHz bandwidth. This was experimentally verified with the disconnected amplifier alone. Excess noise levels in the recorded signals, therefore, must originate from sources other than the amplifier itself.

Thus, the whole circuitry involves only two relevant transistors: one cost-effective IGBT for power switching and one small standard transistor for signal amplification. The critical or limiting elements in this setup are the single IGBT, which will finally fail at excessively high currents or voltages or, in particular, an excessively fast current rise. However, the small transducer coil is endangered by dissipative heat at higher repetition frequencies (for suggestions for avoiding heat or cooling for higher repetition frequencies, see the discussion at the end) and by excessive Lorentz forces (which are, however, the explicit purpose here).

In the concrete realization of the serial power chain (blocks 1–6), all unnecessary inductances except L1 and L2, all undesired resistances except the shunt R1, and all parasitic capacitances to the ground should be avoided or minimized, as they affect the performance and even endanger the IGBT (the parasitic capacitance to the ground promotes a steep current rise). Thus, short, thick, and very direct connections should be established (generously soldered) throughout the serial power chain, which was arranged here on a continuous copper sheet as a common ground plane (Figure 3). When unavoidable, centimeter-wide copper strips served as the interconnection material, instead of more inductive and resistive wire material. From these explanations, it can be easily understood that an almost arbitrary cable connection to the transducer coil notably (but not substantially within the scope of this study) affects the overall performance.

The whole system was not shielded against EMI interference but openly operated in a standard laboratory environment (Figure 3). Therefore, in addition to an EMI emission (irrelevant for an academic study), the sensitive detector system received external interference from other electromagnetic sources in the laboratory environment. However, the external noise was sufficiently weak compared with the ultrasound echoes.

The dynamic of the pulse generation was also modeled with the circuit simulation software LTspice. This tool is readily available for download from Linear Technology Corporation (Milpitas, CA, USA). The computer model allowed a comfortable investigation of the influence from the various components in the power chain (blocks 1–6). A cable connection to the transducer line was modeled as a transmission line (e.g., a coaxial cable) with a specific length (expressed as the delay in nanoseconds) and wave guide impedance. As no ultrasound echo signal was present in this solely electrical simulation, amplifier block 7 was not introduced in this simulation. The simulation results are not shown here, because they reasonably matched the shown real pulse measurements and therefore do not provide additional information for the reader.

Similar to previous work [10,11], a 6 mm diameter aluminum rod that was 30 cm long and a 6 cm aluminum tube served as targets, providing either a longer or a shorter (respectively) delay time for echoes. The 8 mm transducer coil was positioned close to the flat end of the rod or tube, with an effective gap from 0.15 to 1.2 mm (Figure 3). Aluminum metal (with good electrical conductivity and a low mass density) is usually the preferred choice for EMAT development, as high Lorentz forces together with low mass density promote better electroacoustic conversion and higher ultrasound intensities [10]. As a difference from bulk material, a long rod behaves like a dispersive ultrasonic transmission line with distinct ultrasonic propagation modes [12,13], and thus provides strong echo patterns, helpful for basic EMAT development. The coil-only EMAT preferably excites longitudinal ultrasound waves or normally oriented forces [11], and importantly, when the longitudinal wavelength in bulk material is in the order of the rod diameter, a significant reduction in the group velocity is obtained. Elastic expansion in the lateral (or here, radial) direction, even when only longitudinally excited, exerts greater influence here. A detailed analysis of the modes group velocities in thin aluminum rods with contacting piezo transducers was performed and experimentally verified in 1960 by May [13]. The group velocity of the first longitudinal mode was reduced to a minimum of 40% from the bulk velocity for a 6 mm aluminum rod and at 650 kHz. Toward higher frequencies (here, 1–1.2 MHz), the group velocity gradually increases toward 50% of the bulk velocity. A reduction in the group velocity was appreciated in this demonstration study. The decrease increased the delay between the pulse emission and the echo arrival and in this case allowed for a longer recovery time of the intentionally simple echo amplifier block 7.

Thus, the longer rod was used for tests on prolonged time scales up to 1 ms. As a disadvantage, transduction through the longer rod included other higher modes and was notably dispersive. These effects could have been suppressed by tuning the system to much lower frequencies (say 250 kHz), where only the first longitudinal mode and low dispersion were present. However, it seemed more convincing to stay with a megahertz frequency in this study.

Instead, the shorter (6 cm long) aluminum tube with a 6 mm outer diameter and a 4 mm bore was applied for measurements on short time scales. The bore enforced mono-modal transmission (almost exclusively the first longitudinal mode was excited and observed at 1 MHz) and with less pronounced dispersion. It permitted a statement about the system’s currently available response time, and it much better represented the actual shapes of the transduced ultrasonic pulses.

A detailed investigation of the well-known [13] ultrasound propagation effects (various modes and dispersion) in transmission lines or rods was not performed here, as the approach of this contribution is the principal novelty of the transducer scheme itself.

## 3. Results

### 3.1. Emission Pulses

**Figure 4:**
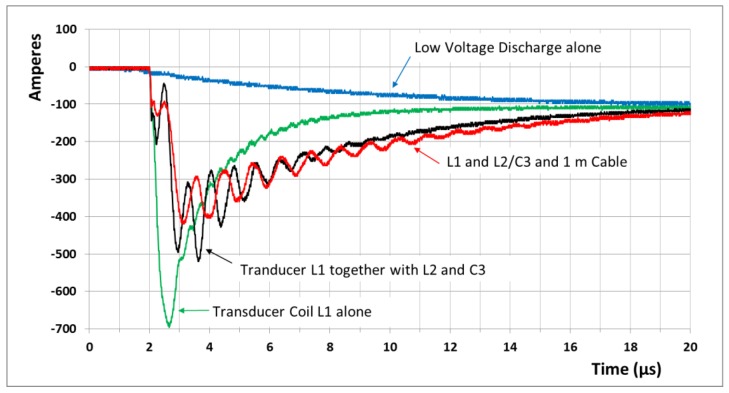
The measured currents on the microsecond-scale approach several hundred amperes immediately after switching. Function block 5 (L2 and C3) imprints a distinct 1.2 MHz modulation in the current pulse, directly effective for ultrasound emission. After 20 µs, the current decays to an almost constant “DC” value of about 100 A, fed from the low-voltage bank block 3. A low-voltage slow discharge alone, without contribution from the high-voltage bank 2, is shown for comparison. With an additional cable connection, the modulation frequency and the peak amplitudes are reduced to 1.05 MHz and 400 A.

Figure 4 shows the experimentally recorded current pulses on the microsecond scale through the serial power chain, from blocks 1–6 and for different situations. The strongest pulse is obtained for the transducer coil L1 alone, without a cable connection and for the bridged L2 (i.e., function block 5 is not involved here). The current rises within 0.7 µs to almost 700 A and then more slowly falls back to an almost constant value of 100 A. The “DC-like” 100 A is fed from the low-voltage capacitor C2. The transient and initial magnetic pulse excites an audible ping in the aluminum rod, whereas a low-voltage soft current pulse from C2 alone is barely audible. For comparison, Figure 4 also shows the slow “DC” current exclusively fed from C2 and without a high voltage charge in C1. A much less dynamic increase to about 100 A is obtained.

Without function block 5, echo detection is not possible in this circuitry. Furthermore, the insertion of L2 and C3 changes the current pulse: in addition to the reduced maximum amplitude (500 A), a considerable 1.2 MHz ringing is also impressed into the initial pulse. It is clear that this unipolar MHz burst (=strong DC field + strong AC field, see, e.g., Ref. [11] and the corresponding supplement) directly translates into 1.2 MHz ultrasonic excitation of the aluminum target. The corresponding echo will be discussed.

Shown in Figure 4 is a current signal with block 5 through a 1 m connection cable between the circuitry and the transducer coil. The amplitudes are further reduced to about 400 A, and the imprinted modulation is reduced in amplitude and frequency to about 1 MHz. The reduction was caused by the additional inductance of the cable connection. These experimental emission pulses can be reasonably reproduced with a simulation in LTspice. However, the actual endurance at critically high powers along with the ultrasound transduction and echo signals are not readily predictable with this software tool.

### 3.2. Echo Signals on Long and Short Time Scales

**Figure 5:**
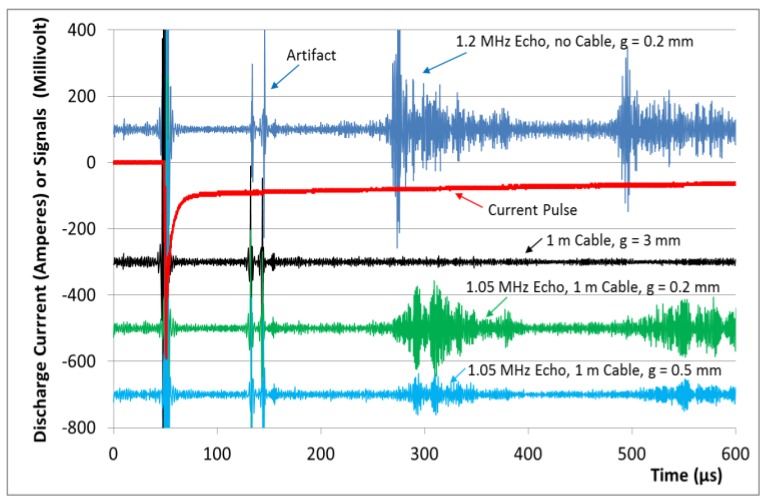
Characteristic current pulse and recorded ultrasound echoes from a single pulse (with numerical offsets for better representation) from the 30 cm aluminum rod and over a prolonged time period (600 µs). The frequencies of the echo signals cannot be recognized in this time scale; however, the actual periodicity of the echoes is closely related to the imprinted modulations in Figure 4. After quick relaxation of the intense excitation pulse, the 100 A “DC” bias current persists much longer, over hundreds of microseconds. The amplifier for the echo signal requires recovery time and relaxation until about 100 µs after the power pulse (artifacts at 120–160 µs). After 220 µs and 440 µs, a distinct first and second 1.2 MHz echo from the aluminum rod (dark blue: distance g = 0.2 mm and no connection cable used) is obtained, with amplitudes of up to 300 mV. A weaker (green) and (due to the dispersion of the group velocity in the aluminum rod) somewhat delayed echo pattern with about 1 MHz is obtained when a 1 m connection cable is used. The coupling efficiency of EMATs is strongly affected by distance: when the gap g between the aluminum rod and the transducer coil is increased to g = 0.5 mm or g = 3 mm (light blue and black), the echo signal is significantly reduced or even fades away.

Figure 5 is based on a prolonged time scale and utilizes the 30 cm dispersive aluminum rod. The figure shows the measured current at the 1200 V charge and the received echo signals from a single pulse (a non-averaged single event), with and without a cable connection and with the required block 5. Offsets of 400, 600, and 800 mV were subtracted from the signals through the cable for a better presentation. Digital bandpass filtering from 0.5 to 2 MHz was applied to the raw data. Multiple echoes (ultrasound pulse traveling several times back and forth in the aluminum rod) are detectable after the 100 µs recovery and relaxation of the detector circuitry. The echoes contain the preferred emission and detection frequency (1.2 MHz or about 1 MHz with the cable, not visually solvable in this prolonged time scale) from the system. The first echo with remarkable amplitudes up to 300 mV is recorded after 220 µs, and this delay would result in a speed of sound in the order of (2 × 0.295 m/220 µs) = 2680 m/s for the aluminum rod, which is significantly less than the speed of longitudinal sound in aluminum bulk material (6250 m/s, [12]). The thin 6 mm aluminum rod does not behave like bulk material, as an ultrasonic wavelength at 1 MHz is already in the order of the rod’s diameter, and the group velocity of the preferably excited longitudinal mode is strongly decreased [13]. The received echoes extend over about 100 µs, instead of representing the approximate 5–10 µs of the excitation pulse (Figure 4). This considerable broadening was mainly caused by dispersion effects in this specific rod and by excitation of additional modes with other group velocities. The broad echoes were not a principal shortcoming of the technique: a virtually non-broadened and mono-modal transduction at shorter time scales is shown in the subsequent paragraph. The long decay time of the 100 A detection current is also noticeable in Figure 5, whereas the excitation pulse (presented in Figure 4) appears almost needle-like in this time scale.

When the transducer coil is connected over the cable, the echo signals weaken (about 50% here), as the additional cable affects the performance. However, more signal weakening is readily obtained by an increased air gap g between the coil and the rod end. The weaker echo patterns in Figure 5 are obtained with a cable and then with a cable and a gradually increased gap g. Notably, the echoes with the introduced cable exhibit an additional delay compared with the simulation without a cable. This can be explained by the gradually reduced frequency with the cable (Figure 4), which in this situation also reduces the group velocity of the propagating wave in the aluminum rod [13]. The ultrasound generation and the echo detection rapidly decline with the increasing air gap g between the coil and the metal target. This is a general and very well-known problem for all EMAT techniques.

**Figure 6:**
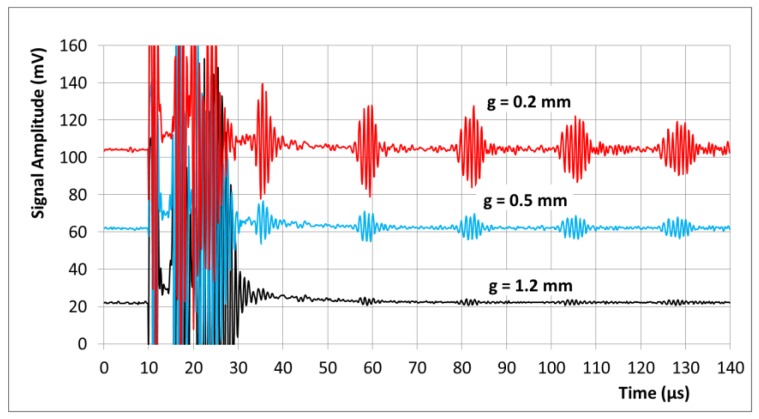
Pulse echo signals (non-averaged) on a shorter time scale. Echoes at 1 MHz are obtained from a mono-modal aluminum tube at different air gaps g between the coil and the target. The transducer coil is operated through a 50 cm thin coaxial cable. The first echo from the 6 cm tube is observed after 20–25 µs: the emission pulse starts at 10 µs, and the maximum of a clear echo is obtained at 35 µs. The early echoes reveal a characteristic width of about 5 µs. A broadening (dispersion of the tube) is observable for the later echoes toward 130 µs. Again, the coupling efficiency and the signal intensity are strongly affected by the air gap, and the echo has become small at g = 1.2 mm. The qualitative signal properties (frequency, time, and width) are virtually not affected by the air gap.

In Figure 6, the system is tuned and evaluated for shorter time scales and in a more pure transduction modality. The short 6 cm aluminum tube with lower dispersion and only one possible longitudinal mode at 1 MHz is used as a target (Figure 3). A much faster recovery and relaxation of the pulse-echo system compared with Figure 5 are obtained by adding several more resistors to the circuit, mainly parallel to the coils L1 and L2 for dampening and parallel to the signal outlet. This dampening in the power chain also shortens the imprinted modulation (Figure 4) to 5 µs. In addition, the 50 kΩ resistor at T2 is replaced with only 5 kΩ for stronger negative feedback and then faster recovery of the amplifier T2. These dampening and stabilizing resistors also reduce the gain and the signal amplitudes from the system. In this experiment (shown in Figure 3), the transducer coil was operated through a 50 cm and 3 mm thin coaxial cable.

Figure 6 shows single pulse events. The signals were not averaged over a pulse series but only bandpass filtered from 0.5 to 2 MHz. The first longitudinal mode is excited along the 1 mm walls of the aluminum tube, and the first echo is observable after about 20–25 µs (the pulse emission starts at 10 µs, and the maximum of the echo is present at 35 µs). The speed of sound approaches the order of at least 4800 m/s in the 6 cm tube, which comes closer to the speed of longitudinal sound in bulk aluminum. A transversal wave travels more slowly (ca. 3100 m/s, [13]) even in bulk aluminum and, therefore, cannot be present here. The length of the echo (FWHM about 5 µs) becomes similar to the emission pulse itself. Thus, the whole bandwidth of the transducer chain is at least 200 kHz here. The relative broadening of the subsequent echoes points to smaller dispersion effects of the tube, in contrast to the very broadened echoes in Figure 5.

The influence of an increased air gap between the transducer and the tube is similar to that shown in Figure 5, the signal strongly decreases with the increasing gap. The gap, however, has virtually no influence on the qualitative appearance of the echoes (arrival time, width, and frequency). This finding supports the thesis of the simple and quasi-hydrostatic coupling of the coil, effective for longitudinal excitation.

The detection of an echo after 25 µs was already close to the principal limit of this simple pulse-echo-system: the powerful emission pulse itself already extends toward 10 µs (Figure 4). What is also important in this representation is that the actual 100 A “DC” bias current is apparently very smooth, as it makes virtually no additional contribution to the noise level. The noise amplitude is similar before (<10 µs, amplifier is continuously recording, but no active signals in the system) and after the switching (>10 µs).

## 4. Discussion

This practical study confirms for the first time the functionality of an intentionally lucid and simple, transistor-controlled, and very compact induction coil without a permanent magnet or separate electromagnet as a contact-less pulse-echo EMAT at about 1 MHz. Due to the strong pulse currents, the initial flux density approaches 4 T at small gaps. This strength exceeds the typically achieved flux densities (in the order of 1 T) of conventional EMATs, to the benefit of the principally available signal intensity. It was shown that the immediate superposition of a strong (here 100 A) and very smooth (no additional noise, Figure 6) “DC” current is possible. A spatially matched bias field was provided and allowed the detection of relatively very weak echo signals with a simple and single stage transistor amplifier. The absence of an additional bulky permanent magnet (which tends to attract ferromagnetic particles and is heat sensitive [2]) or a bulky electromagnet might allow new EMAT applications for narrow and hostile environments. Furthermore, more arbitrary and advantageous coil geometries or designs are possible. For example, the coil could be enforced with a ferromagnetic back plate for further increased electroacoustic conversion [14] and subsequently more reach. A several-millimeter-thick iron powder back plate—with high magnetic permeability, high saturation toward 2 T, and low RF losses—could be directly attached to the back side of the simple coil in Figure 1b, and then more electromagnetic energy would be available for the front side, to the benefit of the electroacoustic coupling efficiency and effective for emission and detection. This modification is much more delicate [14] in the conventional EMAT scenario in Figure 1a. An inappropriately arranged ferromagnetic plate between the coil and the magnet could: (a) shortcut and then reduce the bias field from the permanent magnet; and (b) couple the undesired metallic components of the magnet to the inductor.

The demonstration system suffers under low repetition frequencies, limited by the critical heating of the small coil. Nevertheless, in future and somewhat more advanced designs, the prolonged (here, 1 ms) but heat-effective 100 A for the bias field could be applied for shorter periods of time, only windowing the specific moment when an echo signal is expected. As an example, for the measurement in Figure 6a bias current of only 100 µs instead of 1 ms would suffice. In addition, the small transducer coil could be arranged on a ceramic-like substrate with good thermal conductivity. This could even be the iron powder back plate, which could, in principle, halve the current requirement for a given bias field and thus would even decrease the resistive heating in the filaments to one fourth. Furthermore, the whole sensor could be cooled with forced air from the back side, guided through a fine tube parallel to the connecting cable. When considering potential applications in a narrow, hot, and hostile environment, cooling and/or air purging would be mandatory anyway. The author believes that a quasi-continuous filament heating of 10 W can be technically mastered for a small coil, and this would—including all “tricks”—allow for an ultrasound pulse repetition frequency of more than 10 or even 100 Hz.

It appears reasonable that a more mature circuitry with faster recovery and with better impedance matching (or impedance transformation) and better filtering for more timely, more sensitive, and frequency-selective echo signal detection at higher frequencies could be readily applied in the future, certainly further improving the performance toward the standards of currently established EMATs with additional magnets. Such signal amplifiers are principally well-known from conventional EMAT systems; however, they are less intuitive for the purpose of this initial proof of principle with only two relevant transistors. It is likely that the currently achieved dead time of the system (about 20 µs, Figure 6) can be finally reduced to only a few microseconds and then will allow measurements of samples with smaller thicknesses.

In general, this contribution followed the purist approach with a single induction coil for pulse echo operation. This scenario causes the highest challenges and therefore provides room for splitting off partial functions for less demanding or other EMAT techniques. As an example, a clear disadvantage of the single inductor design is the limitation to preferably emit and detect normally oriented motions or longitudinal waves into bulk material (in addition to the Rayleigh waves parallel to the surface [8]), instead of being very effective for lateral body forces or shear waves. It appears plausible, however, that a system with a single coil can be divided into two separate circuits, which then may drive two flat sandwiched coils: one circuit and coil for the dynamic pulse and echo detection and a second circuit and coil for providing a more prolonged bias pulse. In addition to better heat management, such a close arrangement would allow modified field geometries and then can be designed for efficient shear wave transduction with lower propagation speed [12] and thus be more suitable for less thick samples, for example. The second coil for the bias field could be circuited and designed in such a way that the efficiency of the first RF coil is not significantly affected, which then would allow a compact stack or even the integration of the two coils in a single layer, to the benefit of electroacoustic coupling and still allow an advantageous ferromagnetic back plate.
